# A robust fragile watermarking approach for image tampering detection and restoration utilizing hybrid transforms

**DOI:** 10.1038/s41598-025-01297-4

**Published:** 2025-05-21

**Authors:** S. Prasanth Vaidya, Rajesh N. V. P. S. Kandala, P. V. S. S. R. Chandra Mouli, Hatim G. Zaini, Amar Jaffar, Prabhu Paramasivam, Sherif S. M. Ghoneim

**Affiliations:** 1 Department of Computer Science, BVRIT HYDERABAD College of Engineering for Women, Hyderabad, Telangana India; 2 School of Electronics Engineering, VIT-AP University, Vijayawada, Andhra Pradesh India; 3 Department of Computer Science, Central University of Tamil Nadu, Thiruvarur, Tamil Nadu India; 4 Computer Engineering Department, College of Computer and Information Technology, Taif University, 21944 Taif, Saudi Arabia; 5 Computer and Network Engineering Department, College of Computing, Umm Al-Qura University, Mecca, Saudi Arabia; 6 Department of Mechanical Engineering, Mattu University, Metu, Ethiopia; 7 Department of Research and Innovation, , Saveetha School of Engineering, SIMATS, Chennai, Tamil Nadu - 602105, India; 8 Department of Electrical Engineering, College of Engineering, Taif University, Taif, Saudi Arabia

**Keywords:** Digital watermarking, Discrete wavelet transform (DWT), Image tamper detection, Image recovery, Electrical and electronic engineering, Engineering

## Abstract

This study presents a novel fragile watermarking technique to detect and restore image tampering, enhancing security in digital image transmission. The proposed method integrates Schur decomposition and discrete wavelet transform (DWT) for watermark embedding, ensuring robustness against attacks compared to existing methods. Schur decomposition provides numerical stability in matrix factorization, while DWT enhances resilience through multi-resolution analysis. A semi-blind extraction algorithm, relying only on a secret key, enables active tampering detection without requiring the original image. Upon detection of distortions, the proposed recovery mechanism restores the tampered regions of the image. The effectiveness of the proposed scheme is validated through structural similarity, peak signal-to-noise ratio, and normalized cross-correlation metrics, demonstrating superior performance compared to existing methods. This approach is applicable to secure medical imaging, forensic investigations, and copyright protection, ensuring image integrity in real-world scenarios.

## Introduction

Images are crucial in various fields, such as military intelligence and forensic investigations. In contemporary society, most images exist in digital formats, which facilitates their easy alteration using photo manipulation software, often without the user’s prior expertise or knowledge^1^. It is becoming increasingly difficult to tell whether an image is genuine. So, for any investigation, it has become crucial to detect image tampering. The present technology is making the internet more suitable for sharing data, and there is a rapidly increasing use of social media sites like Instagram, Twitter, Facebook, and WhatsApp. The data that is circulating on the internet contains many forms and formats with varying sizes with fake and genuine information^2^. Image forgery refers to intentionally altering content within an image to deceive or manipulate the information presented in the host image. This process can entail modifying specific areas or multiple sections of the image. Various technologies that simplify image modification have emerged in recent years, making image tampering a significant concern. The challenges in detecting such alterations with the naked eye have intensified, enabling falsifiers to exploit these advancements for their purposes^3^. The proposed tamper detection method aims to address this pressing issue effectively.

Watermarking involves embedding digital data into a carrier signal, which may or may not be related to the carrier itself, using methods such as block patterns or direct pixel insertion^4^. This technique is essential for protecting authentic information and validating legal documents. Watermarking-based authentication plays a vital role in tamper detection and recovery and is generally categorized into robust and fragile watermarking. Fragile watermarking is especially valuable for authenticating multimedia content, including images, videos, and audio. Its high sensitivity makes it particularly effective for identifying tampering attempts^5^. In contrast, robust watermarking is designed to endure routine image-processing operations without losing its integrity^6,7^.

**Fig. 1:**
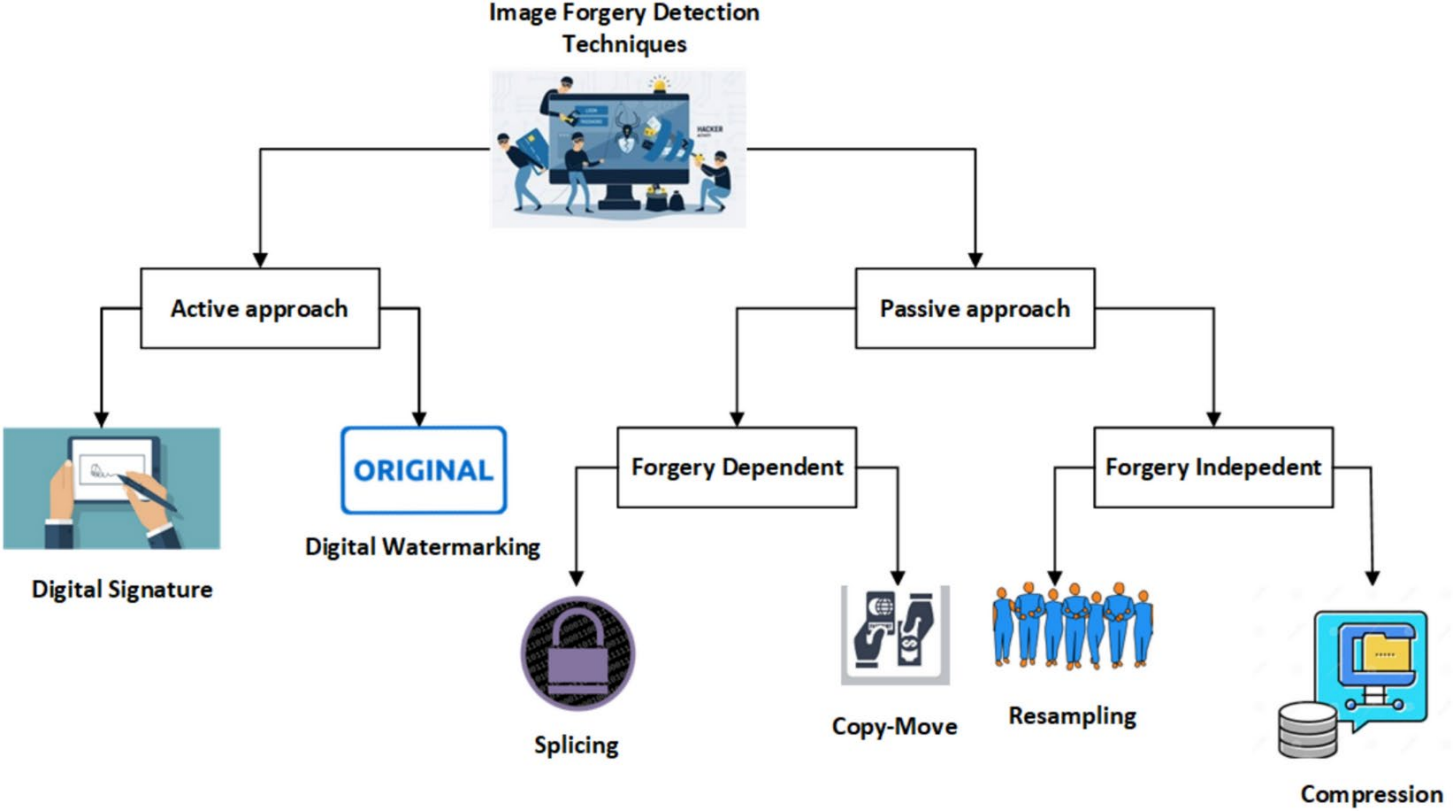
Image forgery classification.

Tamper detection, identifying whether an image has been altered, can be approached in two ways: passive and active^8^. The active approach, including digital signatures and watermarking, involves embedding a watermark or signature into the image during its creation. This embedded information later helps in analyzing any potential tampering. Conversely, the passive approach (blind approach) does not require additional information for forgery detection and relies on features extracted directly from the image. There are dependent and independent methods within the passive approach: the dependent approach focuses on detecting splicing and copy-move forgeries, while the independent approach identifies re-sampling and compression forgeries. This classification of image forgery is illustrated in Fig. 1.

The proposed methodology is designed to detect tampering and recover tampered images using digital watermarking to ensure data security and authenticity. Fragile watermarking is chosen for its high sensitivity, meaning that even the slightest alteration in the image will affect the embedded data. The methodology employs a two-level discrete wavelet transform (DWT) for embedding and extraction. The embedding algorithm uses a secret key for scaling and embedding the watermark. At the first level, DWT decomposes the image into four sub-bands: LL (low-low), LH (low-high), HL (high-low), and HH (high-high). During transmission, the watermarked image may be subjected to various image and signal processing attacks such as copy-move, constant average, cropping, splicing, and noise attacks.

To evaluate the effectiveness of the proposed watermarking technique, metrics such as Peak Signal-to-Noise Ratio (PSNR), Structural Similarity (SSIM), and Normalized Cross-Correlation (NCC) are employed^9^.

The remainder of the paper is organized as follows: Section 2 presents a literature survey on tamper detection and image recovery using watermarking. Section 3 discusses the fundamentals of the relevant methods. Section 4 details the proposed watermark embedding and extraction algorithms, tamper detection, and recovery. Experimental results and comparative assessments are provided in Section 5, and the paper concludes in Section 6.

## Literature survey

In recent years, numerous methods for image watermarking have emerged. Han et al.^10^ introduced a watermarking algorithm using discrete cosine transform (DCT), investigating the relationship between alterations in DCT magnitudes. They employed a Gabor filter to estimate specific image segments, embedding watermark bits based on direction coefficient mapping derived from this relationship, effectively utilizing the direction features of texture blocks. Li et al.^11^ presented a novel approach using synergetic neural networks, processing a significant gray watermark image and embedding it in the block DCT component. Their algorithm used a cooperative neural network to detect and extract watermarks from suspected watermarked signals. Dhaygude et al.^12^ proposed a CNN-based blind watermarking method with an iterative learning framework encompassing three stages: embedding the watermark, simulating attacks, and updating weights to enhance robustness. Singh et al.^13^ proposed a multi-objective medical image watermarking scheme using integer wavelet transform-singular value decomposition for patient data security. Singh et al.^14^ proposed a watermarking scheme using spiral biogeography-based optimization in the wavelet domain.

Almehmadi et al.^15^ developed a method to embed watermarks in Arabic text using counting-based secret sharing. Senapati et al.^16^ combined discrete Tchebichef transform and singular value decomposition (SVD) with scale-invariant feature transform (SIFT) for watermark encoding and decoding. Bhalerao et al.^17^ focused on detecting image tampering and pinpointing the tampered location using a block-based embedding technique and secure hashing algorithm (SHA-1) for verification. Another method, proposed by NR et al.^18^, combined the chaotic properties of the logistic map with SVD for tamper detection and localization.

Siddiqi et al.^19^ implemented an image forgery detection scheme utilizing DWT and dominant rotated local binary patterns (DRLBP) descriptors. Bansal et al.^20^ began generating a shift vector and estimating a threshold using DCT, then assessing matching blocks and shift vectors in the next phase for artifact detection. Abdelhakim et al.^21^ used DCT to embed watermarks, dividing pixels into groups for recovery and applying k-means clustering for image restoration. Rakhmawati et al.^22^ used a block authentication scheme in the spatial domain to generate significant and recovery bits, identifying and restoring modified blocks.

A blind recovery based on integer wavelet transform (IWT) is proposed in Ref.^23^. The proposed method detects the tempering using the check bits inserted in the least significant bits (LSBs). One of the contributions of this work is color image processing, which is not often used in the literature. In Ref.^24^, the authors proposed a semi-fragile watermarking scheme for tamper detection recovery. They used the IWT scheme to generate authenticated watermarks and DCT to recover watermark generation. Later, they tested the tempering using their proposed method on several images and obtained good results in terms of the performance metrics. Hui et al.^25^ proposed a medical image tampering detection algorithm using the texture degree of the medical images and cross-embedding. First, they separated the non-region of interest (NROI) from the region of interest (ROI) in the given medical images. Later, they generated authentication watermarks in ROI to improve the accuracy of the tampering detection. Recovery watermarks were embedded in NROI to recover the images at the destination in the transmission. Durgesh et al.^26^ presented a self-embedding block-wise fragile watermarking for image authentication and tampered area localization and recovery. In this method, the host image is first divided into non-overlapping blocks of size $2$ pixels. Later, for each block, 10 restoration bits and two authentication bits are computed using the five most significant bits of the image. Two-part blocks were also used for the embedding and restoration of that block. It ensures the sure recovery. This process also provides authenticity as the blocks also contain the authentication bits. Using the three-level tampering localization and detection, the algorithm efficiently identified the tampering.

The existing fragile watermarking techniques often suffer from high computational complexity due to neural networks, chaotic systems, or multiple transforms (DCT, SVD, CNN), making them unsuitable for real-time applications. In addition, some approaches lack resilience to geometric distortions, noise, and compression, leading to ineffective detection of tampering and recovery of watermarks. The proposed method aims to develop a secure and computationally efficient fragile watermarking scheme. We used Schur decomposition and DWT for accurate tamper localization and image recovery. We use the authentication block bits (ABBs) to facilitate image recovery and trace collection. By comparing stored LSBs with ABBs, the method enables accurate localization of tampered regions and subsequent restoration, achieving high PSNR values for both watermarked and recovered images. The semi-blind extraction process ensures secure authentication without requiring the original image, making the approach highly effective for secure digital image transmission.

## Methodos used

In this section, we discuss the methods employed in the proposed scheme.

### Schur decomposition

Schur Decomposition (SD) is a technique where a matrix $A$ is decomposed into two matrices $U$ and $\lambda$, such that $A = U \lambda U^{T}$, where $\lambda$ is an upper triangular matrix and $U$ is a unitary matrix. The matrix $U^{T}$ represents the inverse of $U$. In this decomposition, real eigenvalues are positioned along the diagonal of $\lambda$, while complex eigenvalues appear in $2 \times 2$ blocks. The computational complexity of SD is $\frac{8}{3}N^{3}$ floating-point operations (flops), which is significantly lower than the approximately $11N^{3}$ flops required by Singular Value Decomposition (SVD).

The primary purpose of employing SD in calculating Authentication Block Bits (ABB) is to validate each block independently with 16 authentication bits. The image is divided into 128 $\times$ 128 blocks, each size 4 $\times$ 4, and SD is computed for each block. These individual block signatures serve as the ABB for each block. By evaluating each block separately, SD enhances tamper detection accuracy using ABB. The Schur Decomposition for matrix $A$ is expressed as $A = U \lambda U^{T}$. The SD for matrix *A* is given in Eq. 1.

1 $$\begin{aligned} A= U \begin{bmatrix} \lambda _{1}& * & * & *\\ 0 & \lambda _{2 }& * & *\\ 0& 0& \ddots & *\\ 0& 0& 0& \lambda _{n}\end{bmatrix} U^{T} \end{aligned}$$

SD is used for its numerical stability and efficiency in matrix factorization, making it ideal for transforming matrices into simpler upper triangular forms without losing essential image properties. This decomposition enhances the robustness of the watermarking process, ensuring accurate watermark extraction and recovery, even under tampering or transmission distortions. Its integration significantly boosts the scheme’s resilience, making it a vital element in improving the overall reliability of the proposed method.

### Discrete wavelet transform (DWT)

A 2D-DWT is used to decompose the image into subbands. The high-frequency bands (HL, LH) capture the diagonal details. The approximation band (LL) represents low-frequency components, while the detail band (HH) contains high-frequency components. This domain is challenging to configure and provides a robust defense against attacks-the mathematical details of the 2D DWT^27,28^. The 2D-DWT applies wavelet decomposition on the rows and columns of the image separately. That means the 1D DWT operation occurs individually in the rows and columns. It decomposes the image into subbands at different resolutions and orientations.

For a given image *f*(*x*, *y*), two separate 1D DWT will be applied row and column-wise, one after another.

#### Row wise operation

 The first step is to apply a 1D DWT to each image row. This can be represented as:

2 $$\begin{aligned} f_r(x, y) = \sum _{k} f(x, k) h(k - y) \end{aligned}$$

where *f*(*x*, *k*) is the original image, $f_r(x,y)$ is the row-transformed image, and $h(k-y)$ is the filter (kernel) used for low-pass or high-pass filtering.

This operation decomposes the image into low-frequency (approximation) coefficients and high-frequency (detail) coefficients along the rows.

#### Column wise operation

**Fig. 2:**
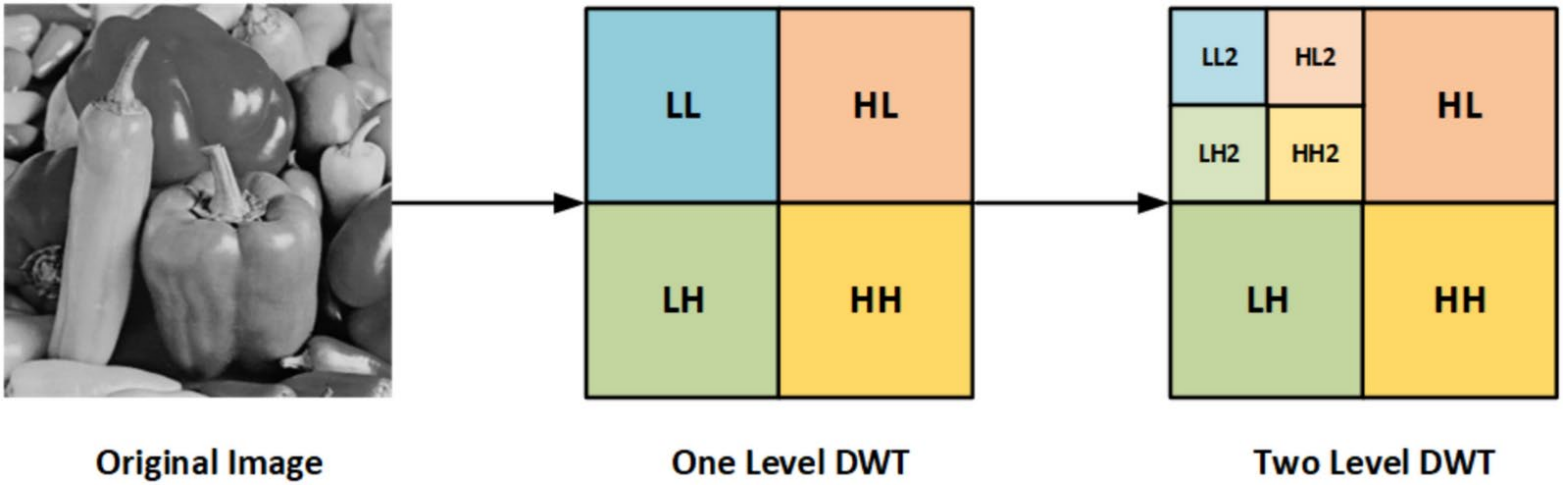
A Two-level subband decomposition of Peppers image using DWT.

 The second step is to apply the 1D DWT along the columns of the row-transformed image:

3 $$\begin{aligned} f_c(x, y) = \sum _{k} f_r(k, y) h(k - x) \end{aligned}$$

where, $f_r(k, y)$ is the the row-transformed image, $f_c(x, y)$ is the column transformed image and $h(k - x)$ is the filter operated along the columns. This operation again decomposes the image into low and high-frequency components along the columns. As mentioned above, after applying this 2D DWT to the image, it will be decomposed into four subbands. In the proposed scheme, the Haar wavelet is employed, and the one-level decomposition of the peppers image is illustrated in Fig. 2.

## Proposed scheme

The proposed architecture comprises three essential modules designed to provide a comprehensive solution. In the embedding module, the process begins with embedding the watermark and authentication bits into the image. These authentication bits are crucial for verifying whether the image has been tampered with, while recovery bits are used to restore the image if tampering occurs. The outcome of this module is an authenticated, watermarked image.

In the extraction module, the authenticated, watermarked image is analyzed for signs of tampering. If no tampering is detected, the watermark is extracted for authentication purposes. Should tampering be identified, the recovery module comes into play. This module uses the recovery bits to restore the tampered image. Each module is described in detail.

### Watermark embedding

In this subsection, the watermark is integrated into the host image, resulting in a watermarked image. The algorithm 1 outlines embedding a watermark into a host image using the Haar wavelet and Schur decomposition techniques. It includes steps for decomposing the image, embedding the watermark, and reconstructing the image while ensuring its authentication through the generation of Authentication Block Bits (ABB).

**Algorithm 1:**
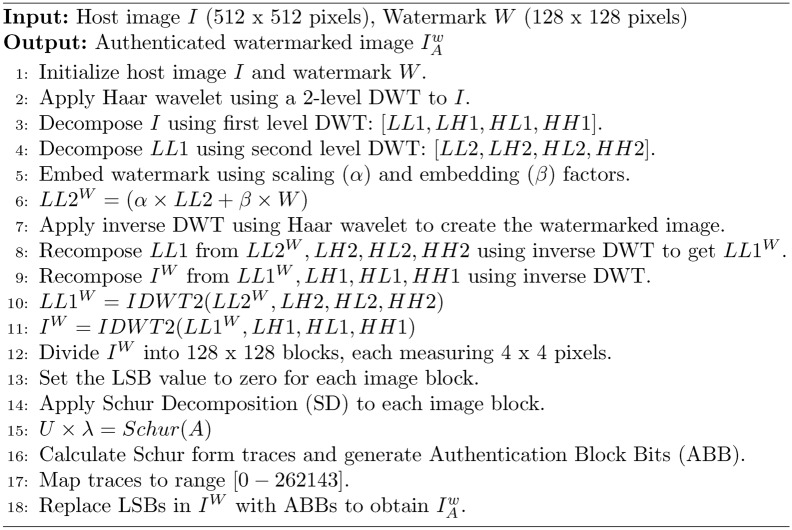
Watermark embedding in host image

**Fig. 3:**
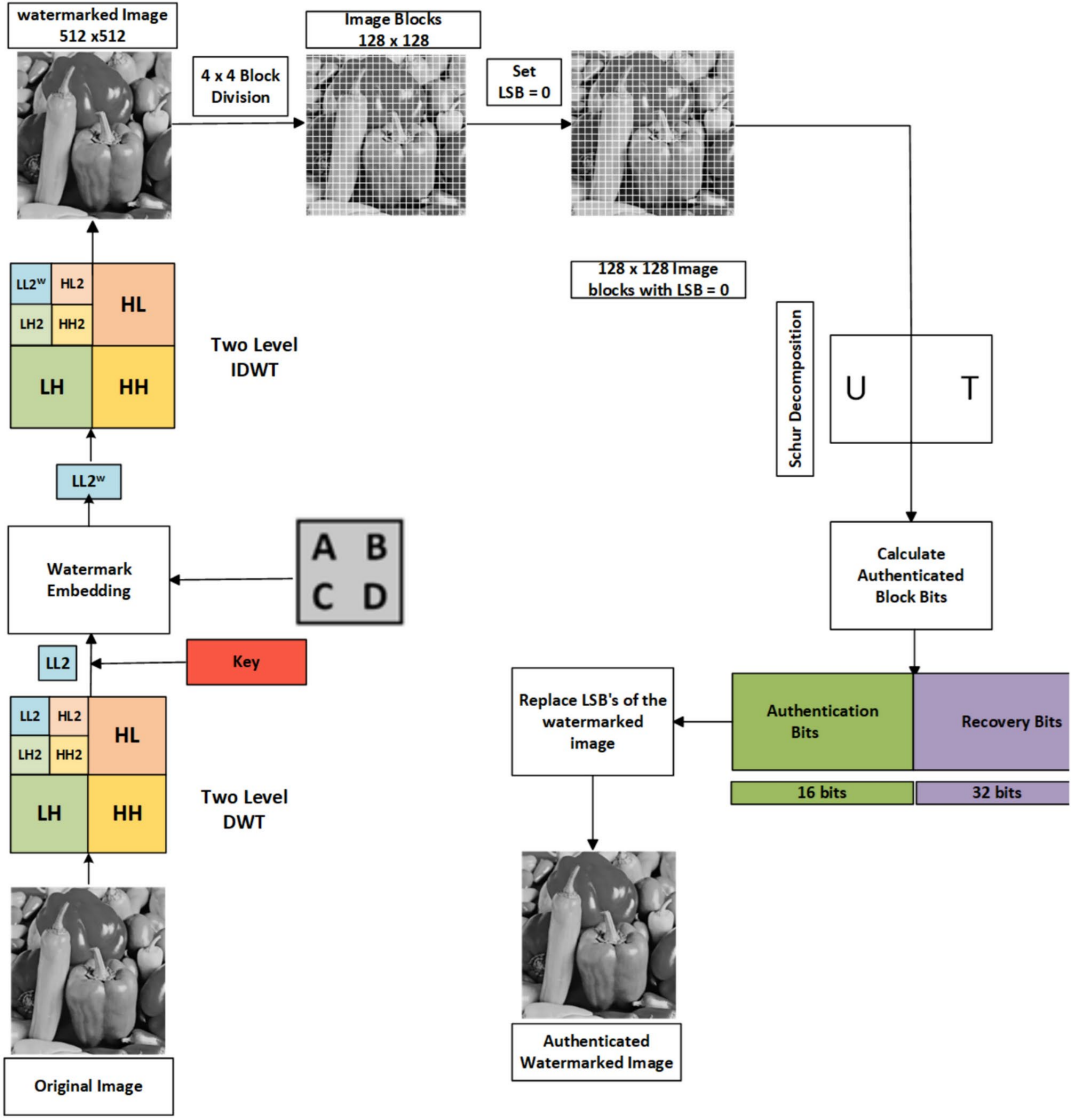
Watermark embedding process generating authentication and recovery bits.

The same is illustrated in Fig. 3. In the watermarking formulae $I_w = \alpha \times I + \beta \times W$, the parameter $\beta$ is known as the embedding factor, which controls the strength and visibility of the watermark *W* in the watermarked image $I_w$. Notably, alpha and beta values are considered Key in both the embedding and extraction processes, as they directly influence the visibility and detectability of the watermark.

### Tamper detection and watermark extraction

**Fig. 4:**
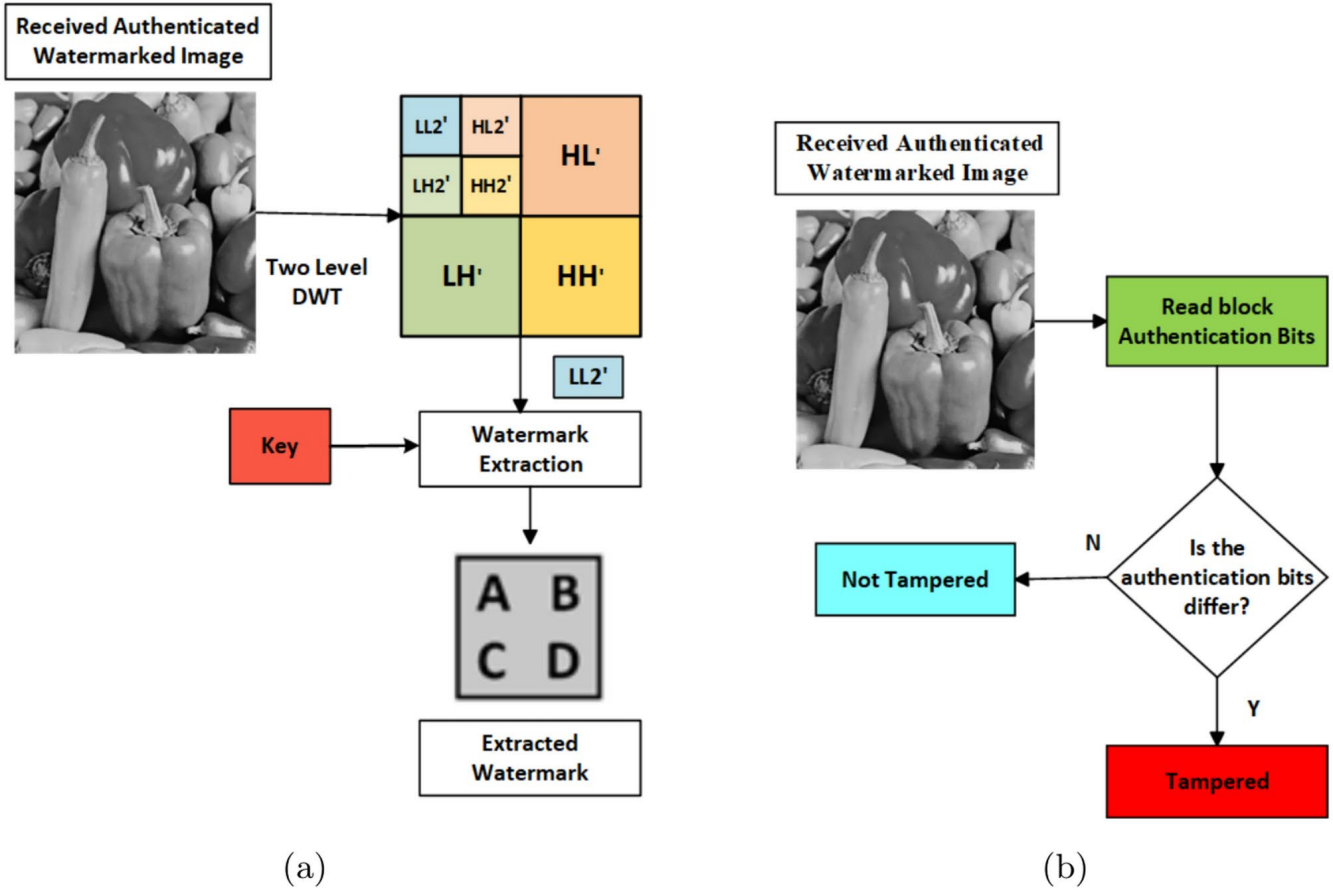
Watermark extraction and tamper detection process. (**a**) Watermark detection. (**b**) Tamper detection.

In this section, the image received by the receiver will be sent to the tamper detection algorithm, and if the image tampers, then it will send a message that the image tampers; else, it will send a message that the image has not tampered, and then the watermark will be extracted. The steps are provided in the Algorithm. 2, and the same is shown in Fig. 4.

**Algorithm 2:**
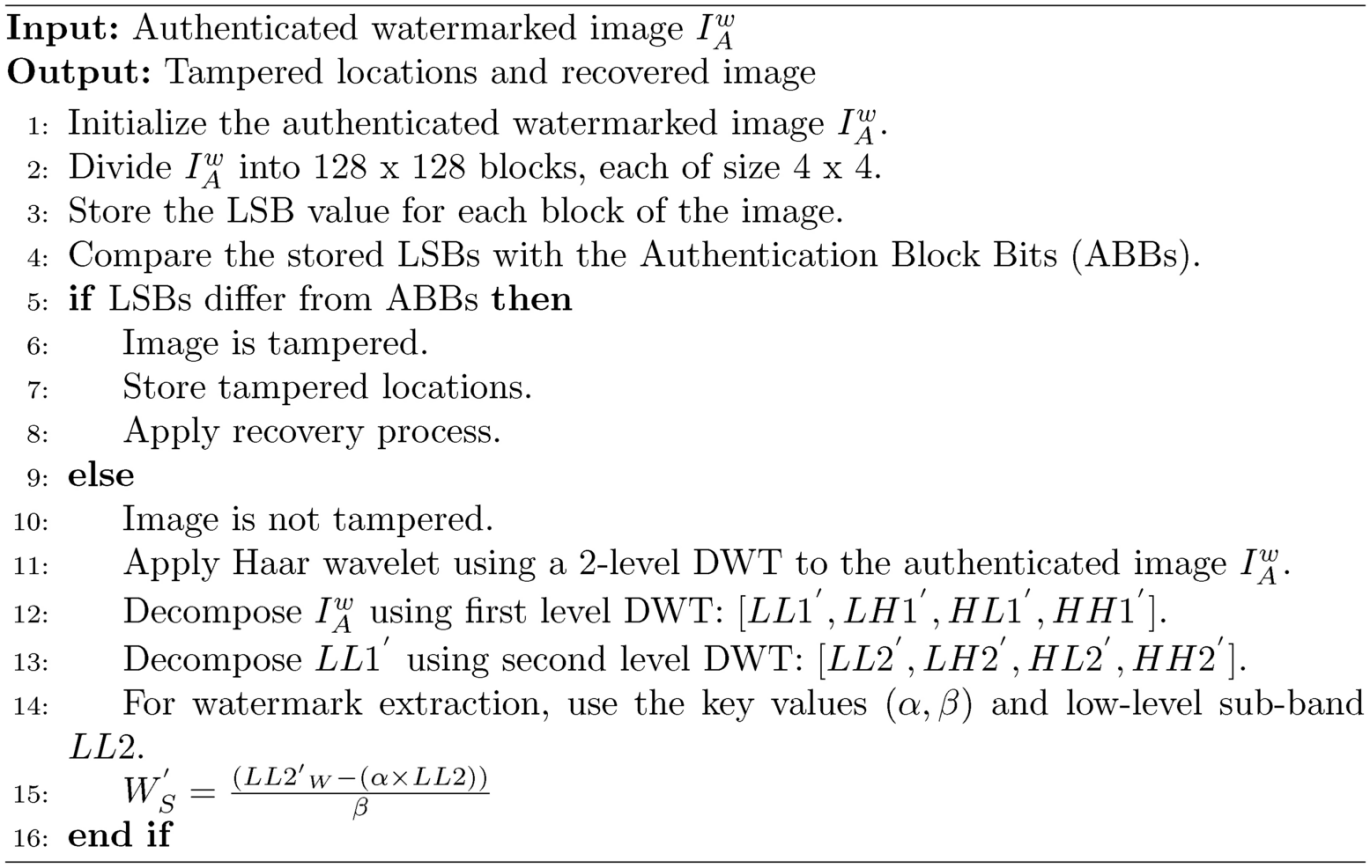
Image tamper detection and recovery

### Tamper localization and recovery

**Fig. 5:**
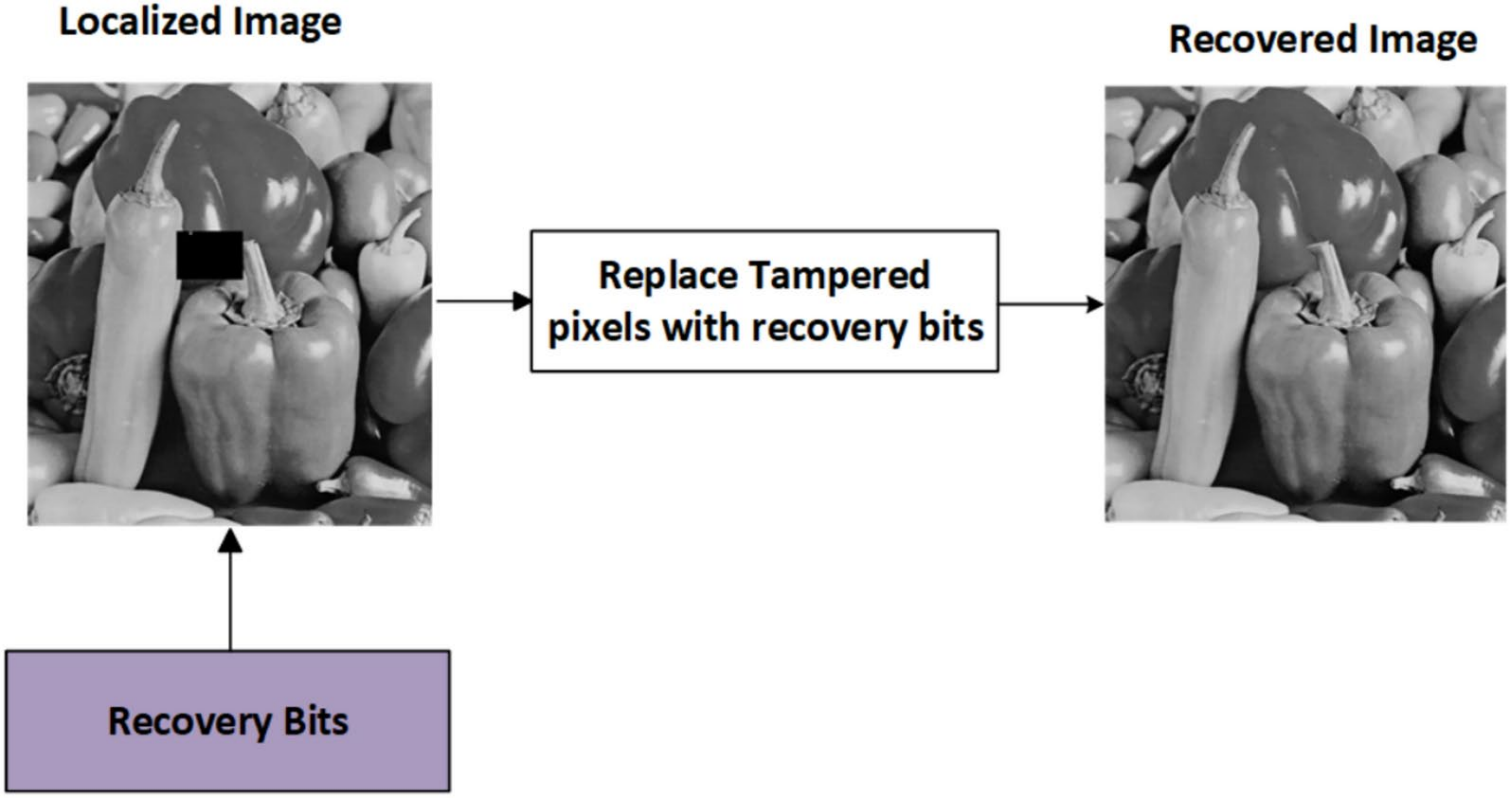
Recovery of image from tampered image using recovery bits.

In this module, the locations of the tampered image are highlighted, and they will be recovered from the tampered image. The recovery process is shown in Fig. 5. The Tamper Localization and Recovery process plays a pivotal role in ensuring the reliability of image content. By effectively identifying tampered regions and applying robust recovery techniques, this method enhances the credibility of digital images, making it a vital component. The Tamper Localization and Recovery module is crucial for identifying and restoring altered regions of an image. The process is outlined as follows:

1. Input Initialization: The tampered image is initialized as the input for the recovery process.
2. Tampered Area Detection: Discrepancies between the extracted watermark and the original image are analyzed to identify tampered pixels.
3. Localized Image Preparation: Tampered pixels in the image are set to zero, creating a localized image that highlights the affected areas.
4. Recovery Bit Generation: Recovery bits corresponding to the tampered locations are generated from the original image, utilizing the information embedded in the watermark.
5. Replacement of Tampered Pixels: The tampered pixels in the localized image are replaced with the corresponding recovery bits, reconstructing the original content.
6. Obtaining the Recovered Image: The recovered image is formed, reflecting the best approximation of the original based on the watermark data.
7. Quality Assessment: The recovery effectiveness is evaluated using the Peak signal-to-noise ratio (PSNR) and Structural Similarity Index (SSIM).
8. Visual Feedback: Tampered areas are visually highlighted on the recovered image, indicating restoration success.

**Algorithm 3:**
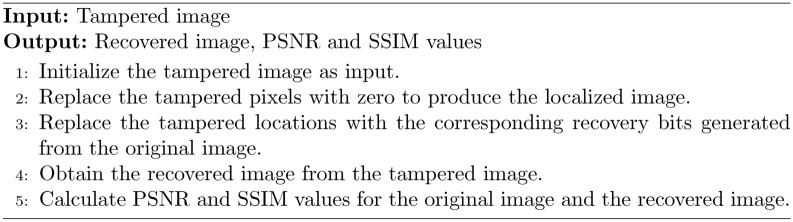
Tampered image recovery

## Experimental results

**Fig. 6:**
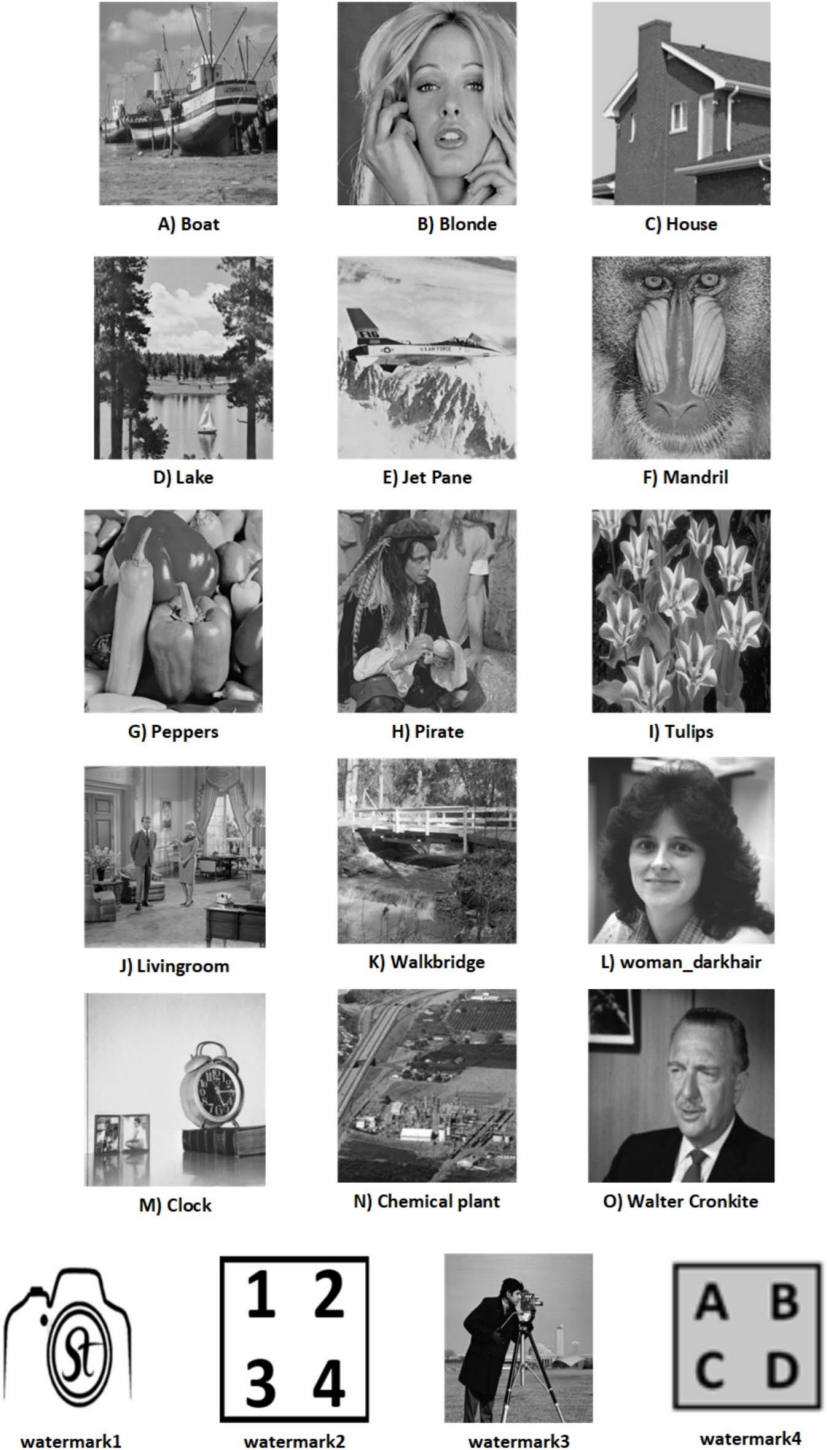
Significant images and watermarks.

**Fig. 7:**
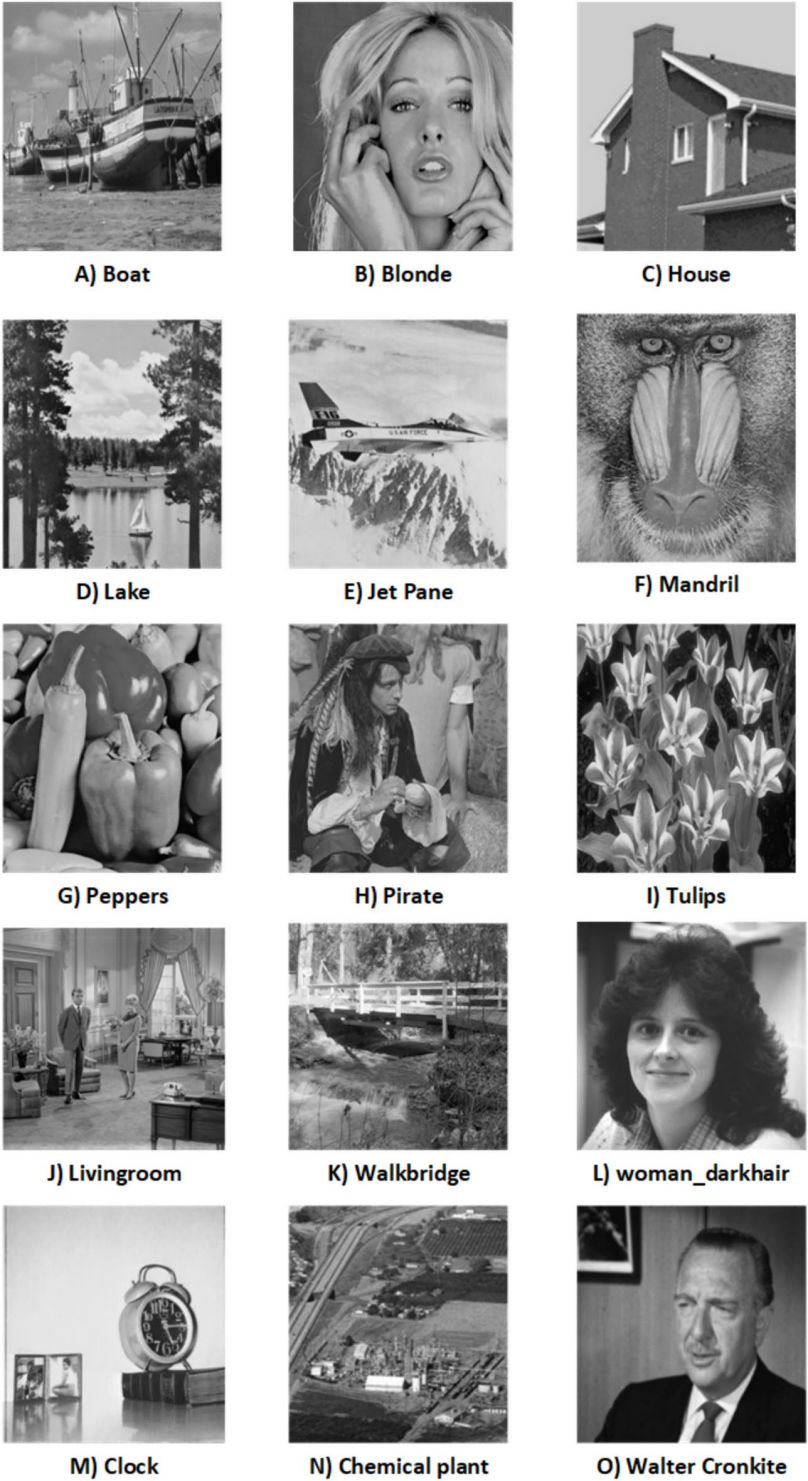
Watermarked images embedded with different watermarks.

**Fig. 8:**
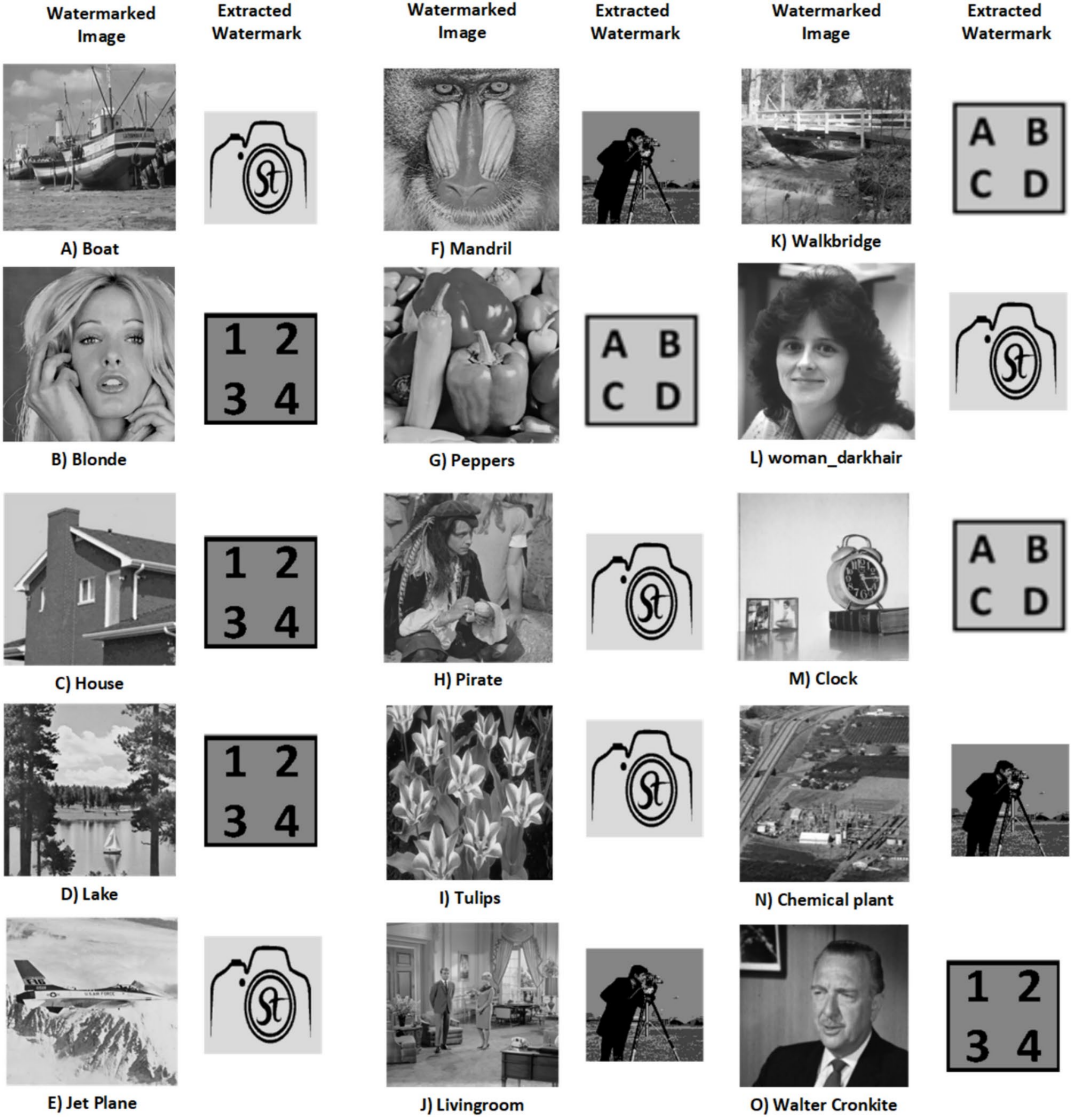
Extracted watermarks from the watermarked images.

The proposed method is evaluated using 15 standard test images from publicly accessible datasets (Imageprocessing Place and SIPI databases)^29,30^. These images, sized 512 $\times$ 512 pixels, serve as the host images, and a 128 $\times$ 128-pixel watermark is utilized. A non-adaptive value of 0.04 was chosen for $\beta$ to ensure that the watermark is embedded in a way that balances visibility and robustness, aiming for a watermark that is detectable but not overly intrusive. In watermarking, $\alpha$ must be chosen such that $\alpha + \beta = 1$. Since $\beta =0.04$ $\alpha = 0.96$. $\beta$ is tested for the range 0.01 to 0.1, and the choice of $\beta = 0.04$ yields the best results, indicating that it strikes an optimal balance between the watermark’s visibility and the preservation of the original image’s quality. Sample images and the watermark are displayed in Fig. 6. The resulting watermarked images are presented in Fig. 7, while the extracted watermarks are shown in Fig. 8. The effectiveness of the scheme is measured using PSNR and NCC metrics.

### Structural similarity

SSIM measures the similarity between the original and watermarked images based on human visual perception^31,32^. The SSIM value ranges from -1 to 1, where 1 indicates perfect structural similarity between the images. It is defined as:

4 $$\begin{aligned} \text {SSIM}(H, H_w) = \frac{(2 \mu _H \mu _{H_w} + C_1)(2 \sigma _{H H_w} + C_2)}{(\mu _H^2 + \mu _{H_w}^2 + C_1)(\sigma _H^2 + \sigma _{H_w}^2 + C_2)} \end{aligned}$$

Where:

$$\begin{aligned} \mu _H&: \text {mean intensity of the original image} \\ \mu _{H_w}&: \text {mean intensity of the watermarked image} \\ \sigma _H^2&: \text {variance of the original image} \\ \sigma _{H_w}^2&: \text {variance of the watermarked image} \\ \sigma _{H H_w}&: \text {covariance of the original and watermarked images} \\ C_1&: \text {constant to stabilize the denominator} \\ C_2&: \text {constant to stabilize the denominator} \end{aligned}$$

The components of SSIM can be expressed as:

Luminance:

5 $$\begin{aligned} l(H, H_w) = \frac{2 \mu _H \mu _{H_w} + C_1}{\mu _H^2 + \mu _{H_w}^2 + C_1} \end{aligned}$$

Contrast:

6 $$\begin{aligned} c(H, H_w) = \frac{2 \sigma _H \sigma _{H_w} + C_2}{\sigma _H^2 + \sigma _{H_w}^2 + C_2} \end{aligned}$$

Structure:

7 $$\begin{aligned} s(H, H_w) = \frac{\sigma _{H H_w} + C_2}{\sigma _H \sigma _{H_w} + C_2} \end{aligned}$$

Thus, the complete equation for SSIM can be represented as:

8 $$\begin{aligned} \text {SSIM}(H, H_w) = l(H, H_w) \cdot c(H, H_w) \cdot s(H, H_w) \end{aligned}$$

### Normalized cross correlation (NCC):

NCC evaluates the similarity between the extracted and original watermark^33,34^. It is given by:

9 $$\begin{aligned} \text {NCC} = \frac{\sum _{a=1}^{m} \sum _{b=1}^{n} w(a, b) \cdot w_e(a, b)}{\sqrt{\sum _{a=1}^{m} w(a, b)^2} \cdot \sqrt{\sum _{a=1}^{m} w_e(a, b)^2}} \end{aligned}$$

In this equation,

w(a,b) and $w_e(a,b)$ represent the pixel values at coordinates (a,b) for the original and extracted watermarks, respectively, while m and n denote the image dimensions.

### Tamper detection rate (TDR)

The Tamper Detection Rate (TDR) measures the proportion of tampered pixels that are correctly identified compared to the actual number of tampered pixels^17^.

10 $$\begin{aligned} \text {Average TDR} = \frac{\text {Number of Detected Tampered Pixels}}{\text {Actual Number of Tampered Pixels}} \times 100 \end{aligned}$$

Table 1 presents the PSNR and SSIM values for the 15 watermarked images, and Table 2 shows the NCC values of the extracted watermarks of the 15 watermarked images with their average.

**Table 1:** PSNR, SSIM values for the 15 watermarked images along with it’s average.

Images	PSNR	SSIM
House	49.30	0.9996
Mandril	45.24	0.9994
Peppers	45.10	0.9987
Lake	49.30	0.9998
Jet plane	41.35	0.9984
Boat	42.12	0.9984
Tulips	43.39	0.9972
Pirate	44.35	0.9983
Blonde	41.11	0.9982
Livingroom	44.25	0.9981
Walkbridge	45.14	0.9991
Woman_darkhair	43.89	0.9988
Clock	45.62	0.9991
Chemical Plant	44.55	0.9985
Walter Cronkite	43.05	0.9991
Average	44.52	0.9987

**Table 2:** NCC values of the extracted watermarks from the 15 watermarked images along with their average.

Images	NCC	Images	NCC
House	0.9881	Blonde	0.9965
Mandrill	0.9610	Livingroom	0.9971
Peppers	0.9408	Walkbridge	0.9976
Lake	0.9881	Woman_darkhair	0.9896
Jet plane	0.9915	Clock	0.9921
Boat	0.9767	Chemical plant	0.9902
Tulips	0.9914	Walter cronkite	0.9934
Pirate	0.9915	Average	0.9857

**Table 3:** Average PSNR, SSIM and NCC values with varying $\beta$.

$\beta$ Values	PSNR	SSIM	NCC
0.02	46.87	0.9991	0.9798
0.03	46.11	0.9989	0.9821
0.04	45.52	0.9987	0.9857
0.05	44.76	0.9895	0.9765
0.06	44.21	0.9826	0.9685

**Table 4:** NCC values of the extracted watermarks from the 15 watermarked images with Salt & Pepper Attack (0.01 to 0.05 Density).

Image/ S & P noise	0.01	0.02	0.03	0.04	0.05
House	0.9321	0.9205	0.8752	0.8526	0.7528
Mandril	0.9489	0.9157	0.8965	0.8517	0.7538
Peppers	0.9453	0.8498	0.8350	0.8025	0.7957
Lake	0.9085	0.8851	0.8587	0.8004	0.7601
Jet plane	0.8908	0.8682	0.8438	0.7961	0.7560
Boat	0.8922	0.8655	0.8319	0.7938	0.7816
Tulips	0.9226	0.8303	0.8343	0.7940	0.7688
Pirate	0.9050	0.8198	0.8010	0.7810	0.7762
Blonde	0.9359	0.8797	0.8569	0.8151	0.7843
Livingroom	0.9259	0.8757	0.8269	0.8051	0.7643
Walkbridge	0.9159	0.8697	0.8169	0.7851	0.7643
Woman_darkhair	0.9359	0.8757	0.8269	0.7951	0.7643
Clock	0.9059	0.8597	0.8269	0.7851	0.7643
Chemical Plant	0.9259	0.8797	0.8369	0.7951	0.7643
Walter Cronkite	0.9159	0.8657	0.8269	0.7951	0.7643
Average	0.8953	0.8867	0.8646	0.8565	0.7923

Table 3 shows the result of PSNR, SSIM, and NCC values over the various values of $\beta$ ($0.02 - 0.06$). The proposed method was tested under a salt-and-pepper noise attack, with noise density levels ranging from 0.01 to 0.05. As shown in Table 4, the scheme demonstrates its robustness by successfully extracting the watermark even at higher noise densities. The experimental results indicate that the scheme maintains high NCC values across all tested noise densities, with all values above 0.75, thus validating its effectiveness. To test the robustness of the proposed scheme, various image and signal processing attacks are applied to sample images. The watermark is extracted from the attacked images, and its NCC is calculated and shown in Table. 5. The Salt & Pepper attack is tested with density 0.01, Gaussian Noise attack with 0 mean and 0.01 variance, Mean and Median filtering of size $3\times 3$, Scaling with 0.5, translating with [2,3] in the *x*-direction and *y*-direction, and finally, cropping 10% of the image.

**Table 5:** NCC values of the extracted watermarks from the 15 watermarked images with Different Image Processing Attacks.

Images/attacks	House	Mandril	Peppers	Lake	Average
S & P Noise	0.9321	0.9489	0.9453	0.9085	0.9337
Gaussian noise	0.8672	0.8511	0.8638	0.8629	0.8613
Mean filtering	0.9387	0.9401	0.9346	0.9336	0.9368
Median filtering	0.9288	0.9249	0.9231	0.9243	0.9253
Rotation	0.8834	0.8895	0.8863	0.8841	0.8858
Scaling	0.9168	0.9114	0.9123	0.9185	0.9148
Translation	0.9347	0.9361	0.9355	0.9313	0.9344
Cropping	0.8725	0.8762	0.8715	0.8796	0.8750

The proposed scheme is tested by applying tampering attacks like copy-move, copy-move mid, splice, and text attacks for various sample images. The explanation of each tampering attack is provided below

1. Copy-move attack: This technique entails selecting a specific area of an image, duplicating it, and pasting it in another location within the same image. This is often used to hide or alter parts of the image. Detection methods focus on spotting similar patterns in the blocks of the image.
2. Copy-move mid attack: This is a variation of the copy-move attack where the duplicated area is modified (resized or rotated) before being inserted, complicating the detection process.
3. Splice attack: In this method, two or more distinct images are combined to form a single misleading image. Detection is based on identifying inconsistencies in lighting and edge transitions.
4. Text attack: This involves altering text in an image, whether by adding, removing, or changing it, to misrepresent the original information. Detection strategies typically analyze the characteristics of the text for irregularities.

**Fig. 9:**
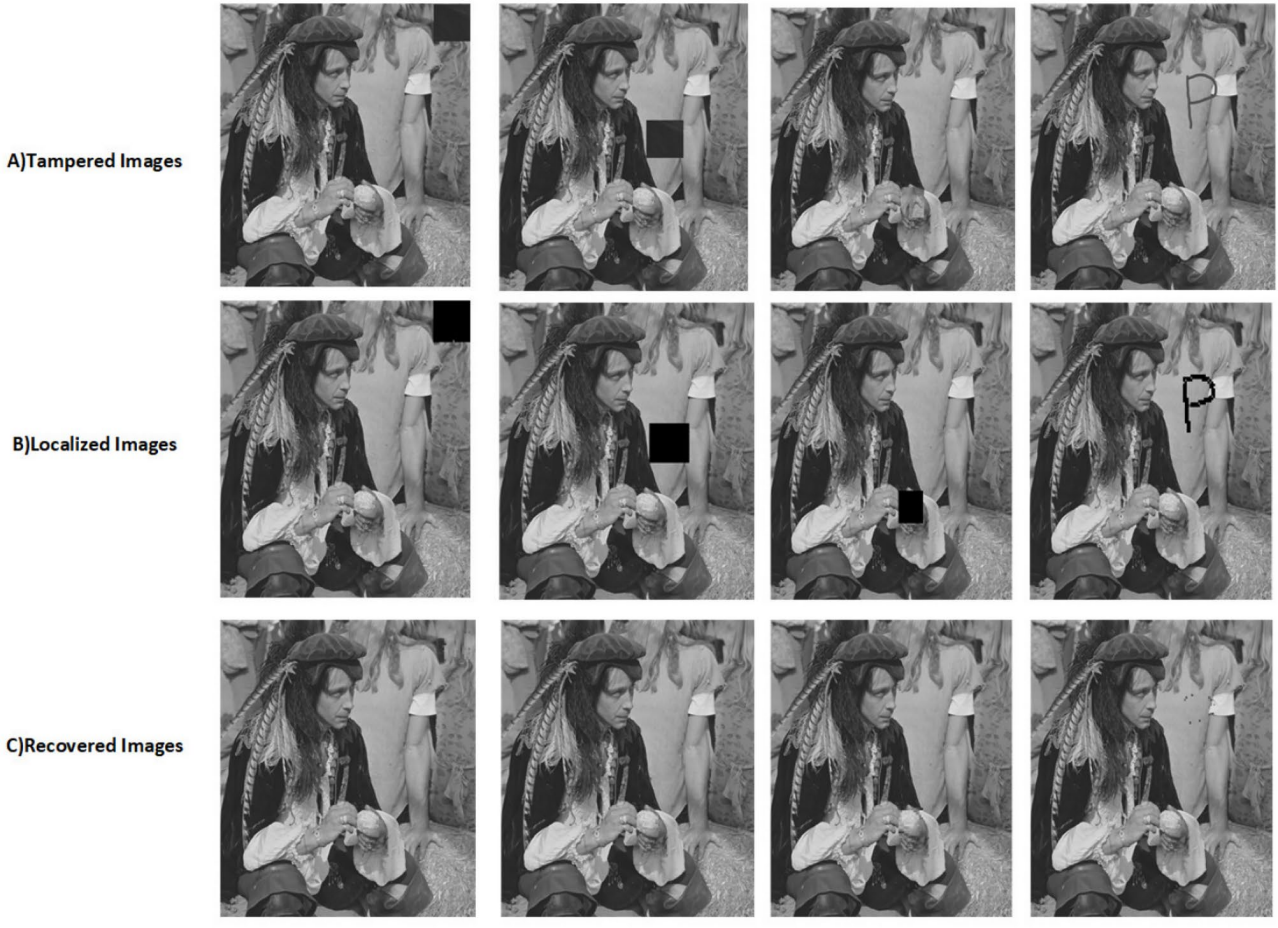
Tampered image, localized image, and recovered image of pirate.

**Fig. 10:**
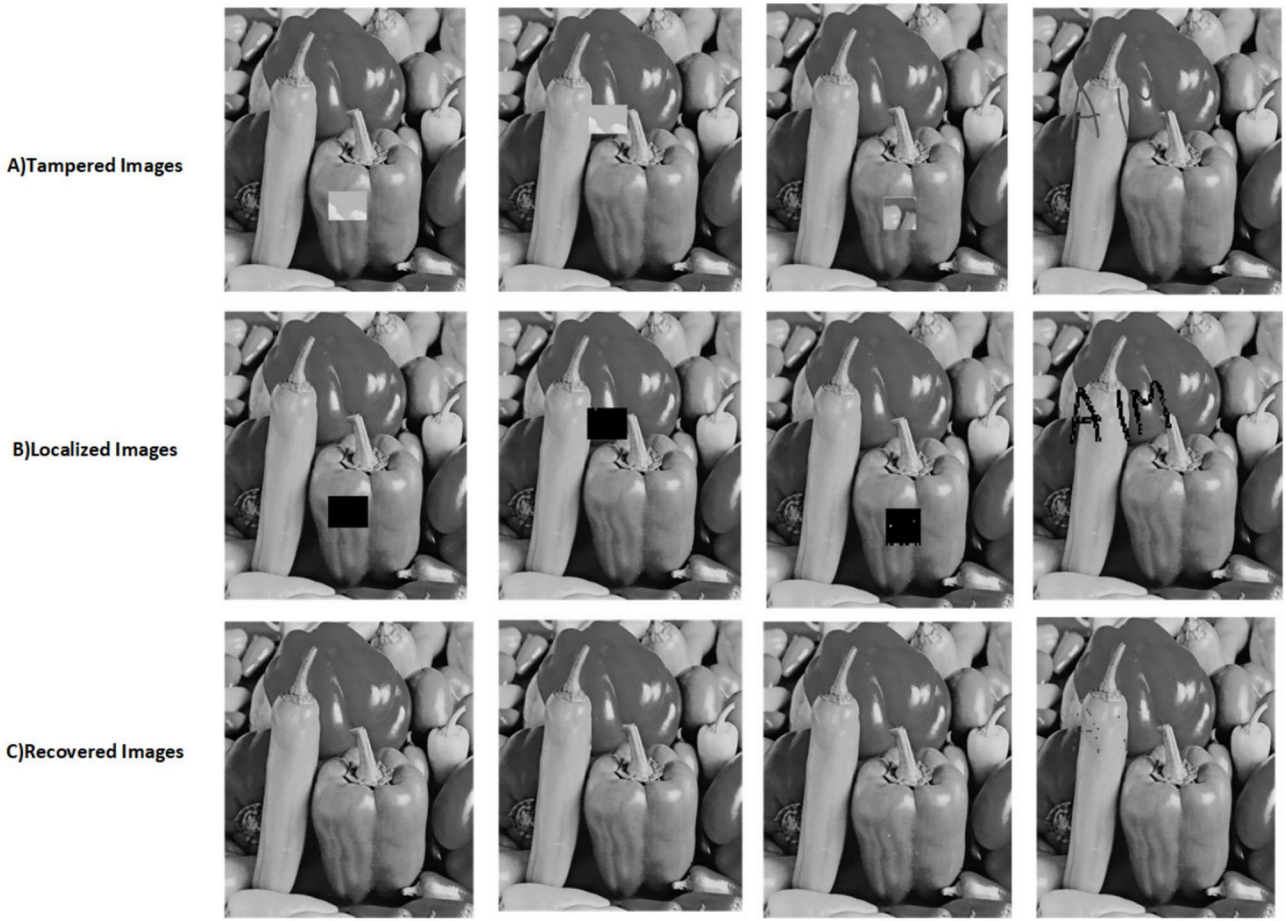
Tampered image, localized image, and recovered image of peppers.

**Fig. 11:**
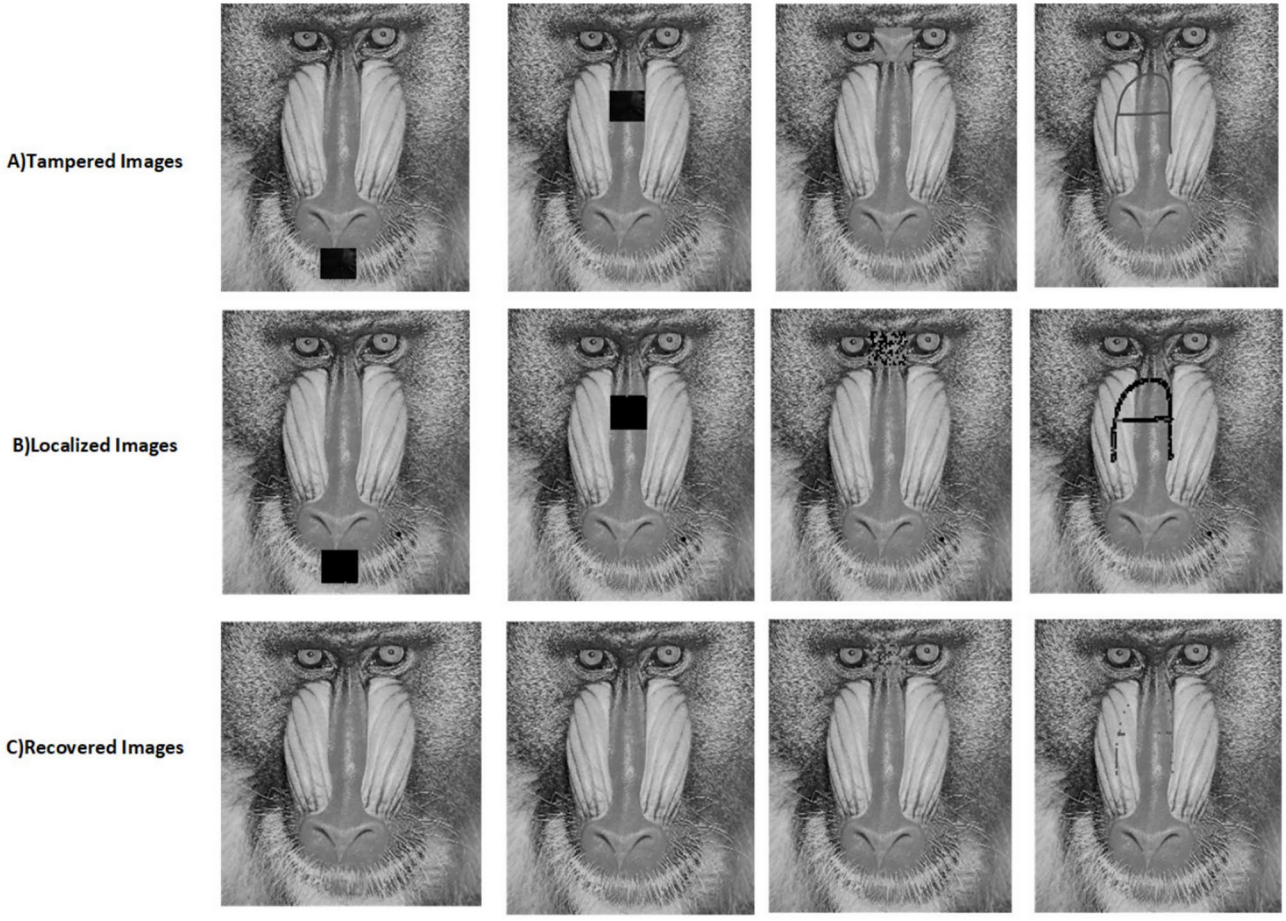
Tampered Image, localized image, and recovered image of mandril.

**Fig. 12:**
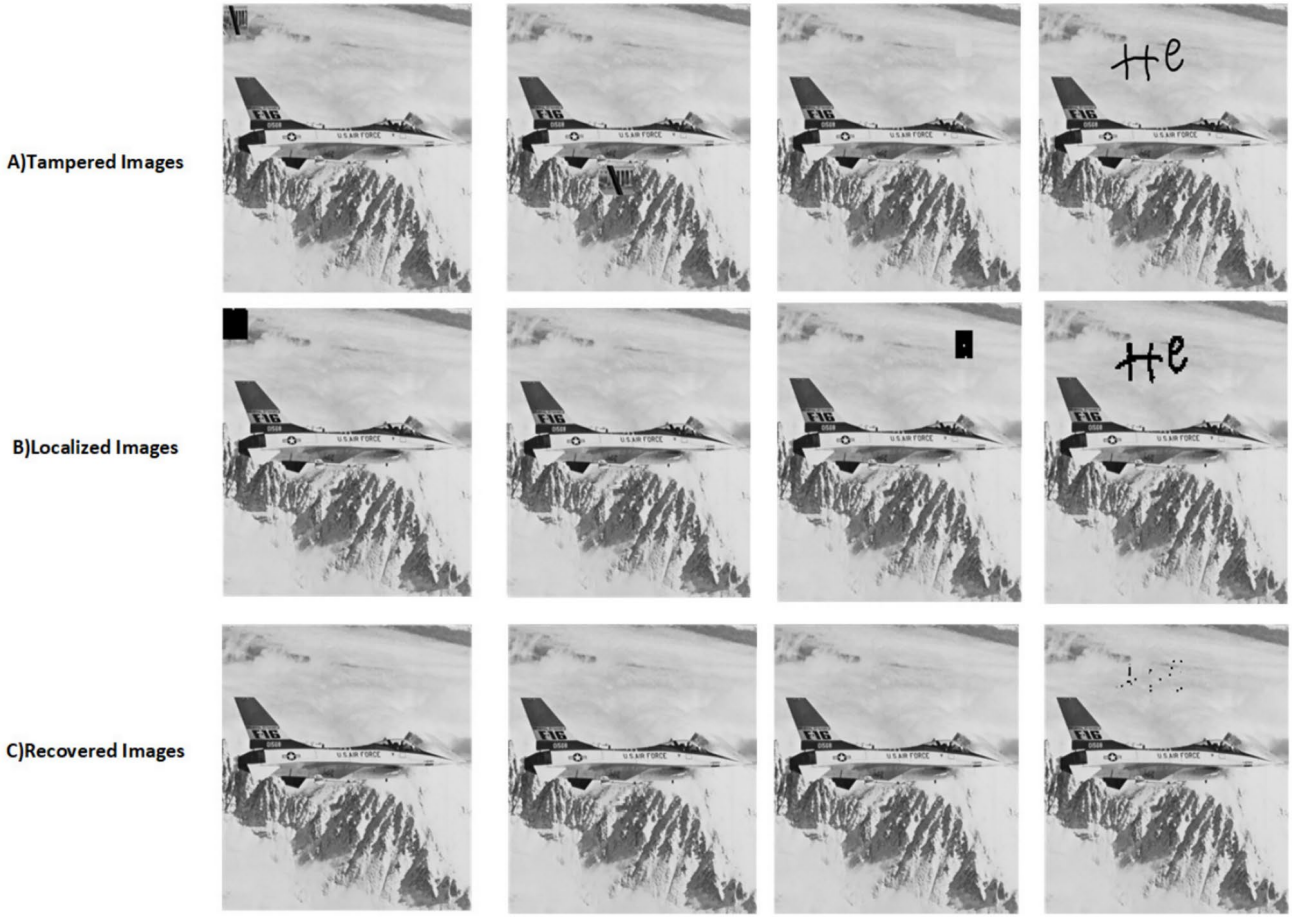
Tampered image, localized image, and recovered image of jetplane.

**Fig. 13:**
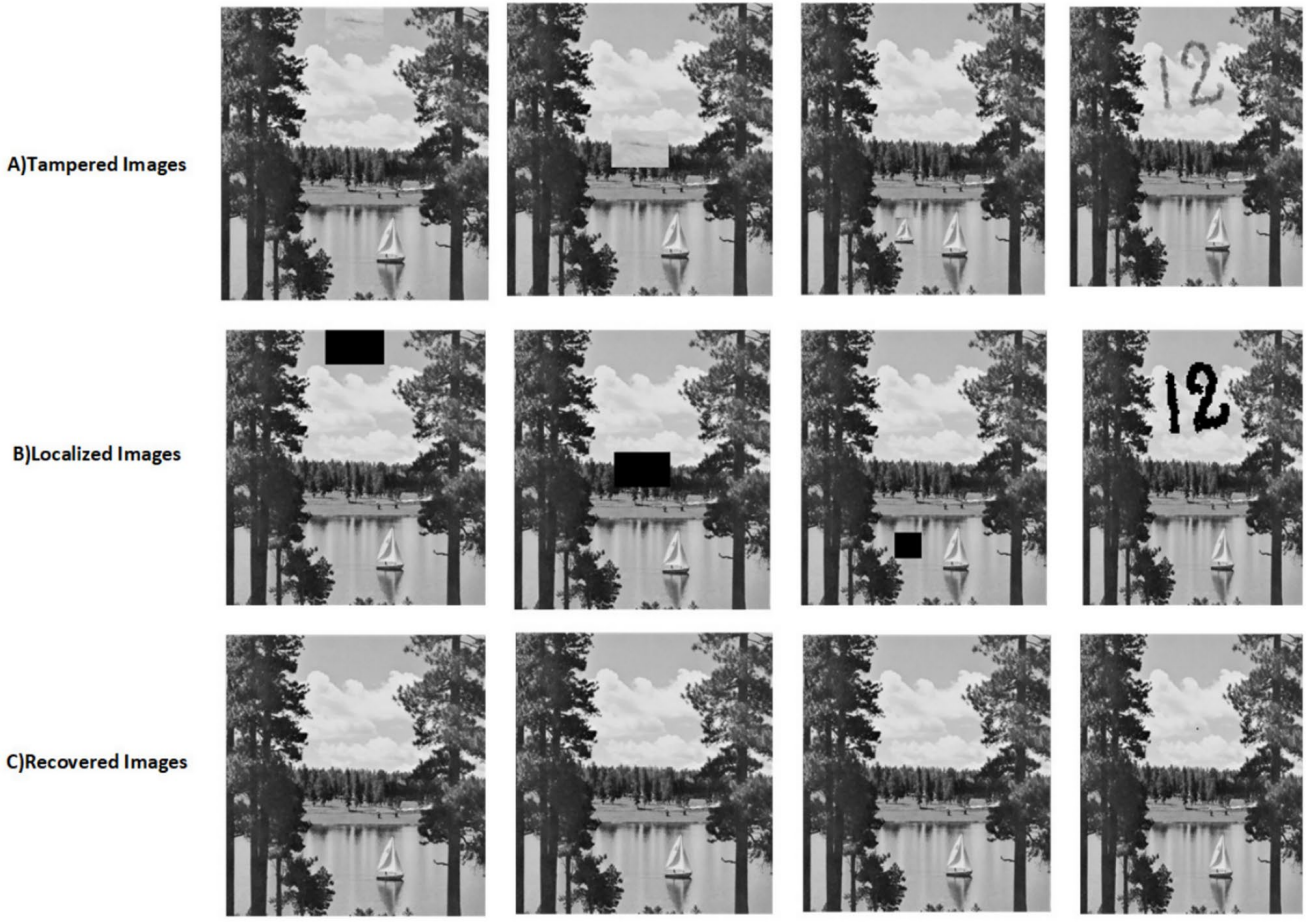
Tampered image, localized image and recovered image of lake.

**Fig. 14:**
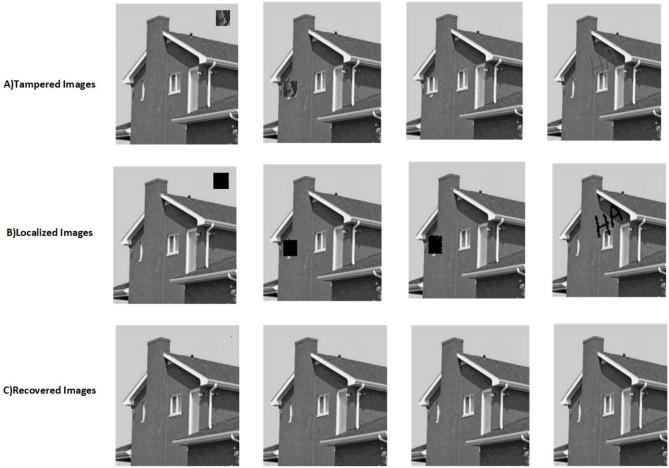
Tampered image, localized image, and recovered image of house.

**Fig. 15:**
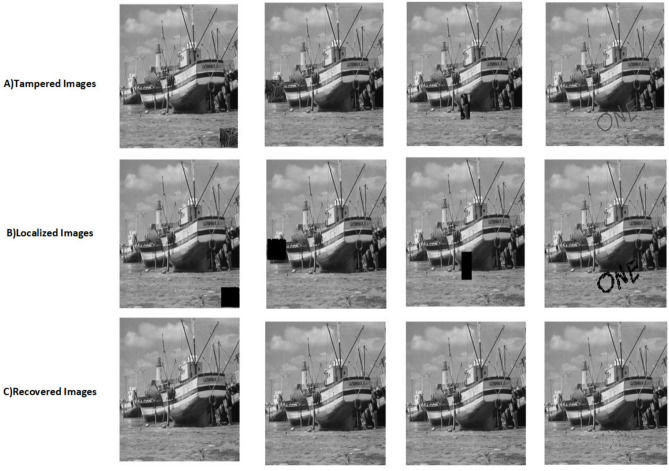
Tampered image, localized image, and recovered image of boat.

**Fig. 16:**
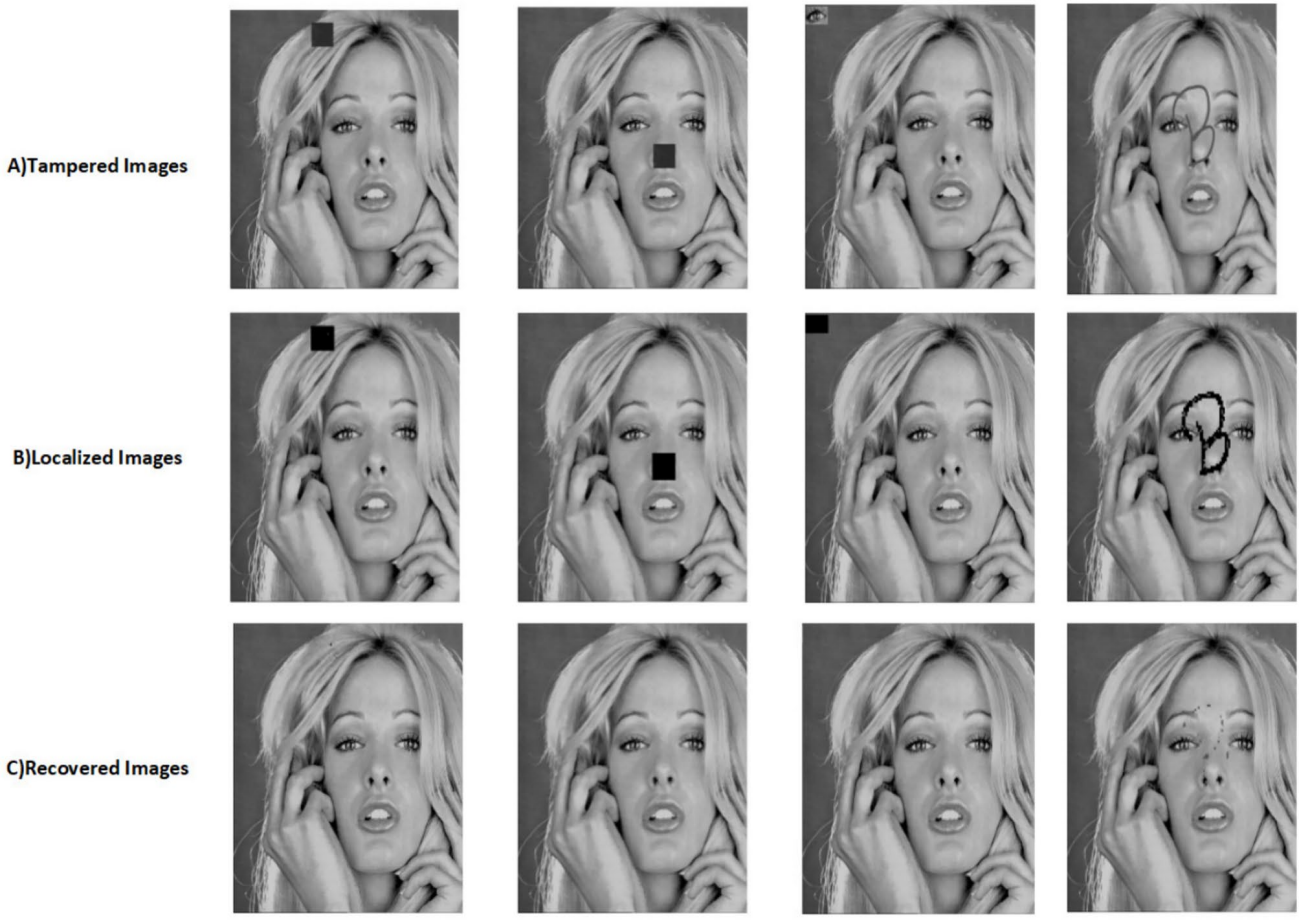
Tampered image, localized image, and recovered image of blonde.

**Fig. 17:**
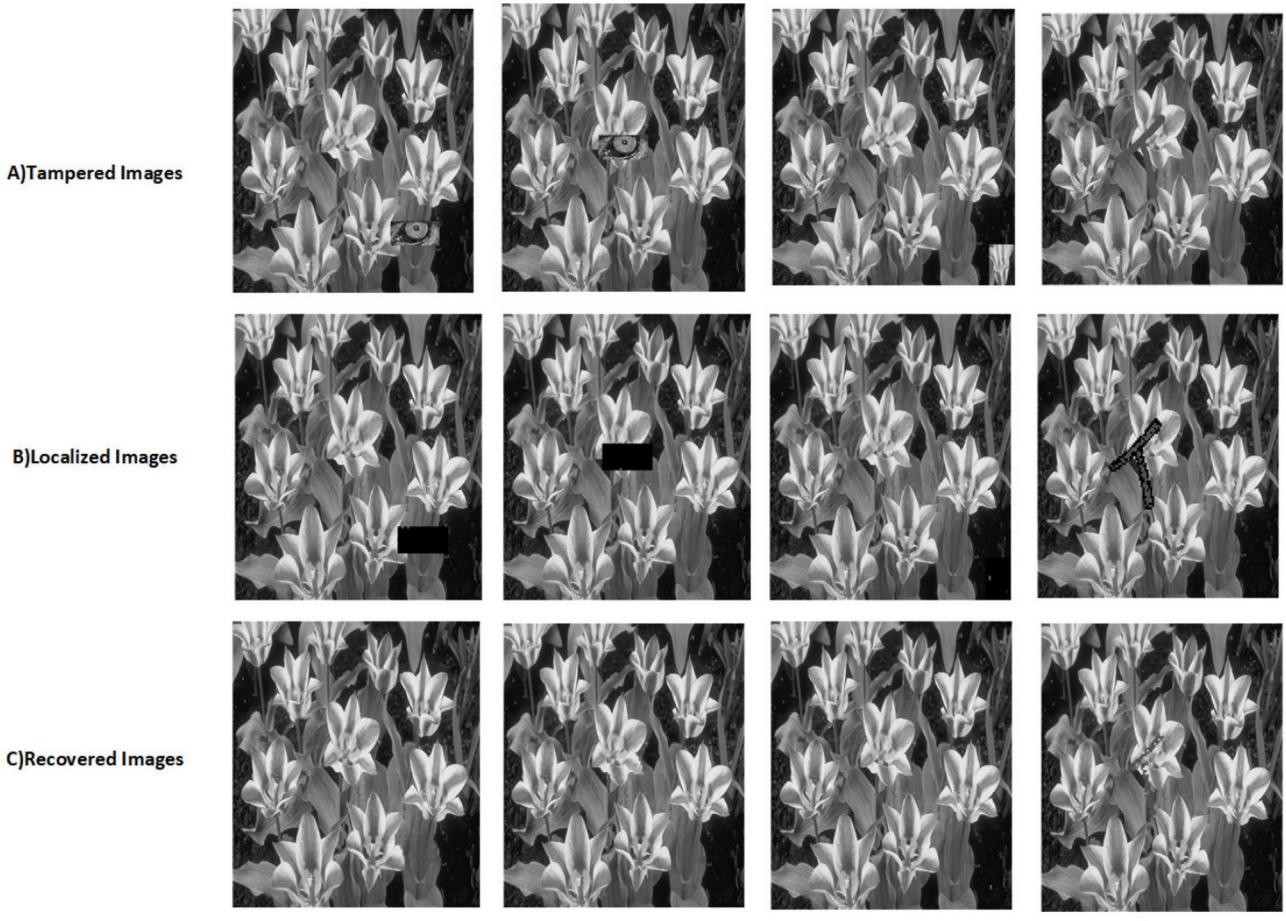
Tampered image, localized image, and recovered image of tulips.

The PSNR and TDR values of the recovered images are calculated to test the image quality, which is provided in Table 6. It can be observed that the proposed scheme can detect 100 % of tampering. The localization of the tampered images and the recovery of the original images for all the sample images are provided. The sample images have tampered in different positions and then the images are localized and then recovered with the proposed scheme are shown in Fig. 9 for pirate, Peppers in Fig. 10, Mandri in Fig. 11, Jetplane in Fig. 12, Lake in Fig. 13, House in Fig. 14, Boat in Fig. 15, Blonde in Fig. 16 and finally Tuplips in Fig. 17.

**Table 6:** PSNR values of recovered image with various tampering attacks.

Images	Copymove	Copymovemid	Splice	Text	TDR
House	46.60	42.12	41.79	44.39	100
Mandril	38.42	42.09	31.21	35.57	100
Peppers	44.14	40.84	42.85	37.99	100
Lake	49.04	38.09	49.65	45.81	100
Jet plane	39.98	36.19	40.62	31.32	100
Boat	38.51	34.29	38.49	36.23	100
Tulips	37.80	35.87	38.72	31.88	100
Pirate	44.76	45.47	47.84	50.95	100
Blonde	48.87	49.39	49.90	47.84	100
Livingroom	47.26	46.35	45.02	45.33	100
Walkbridge	46.58	42.38	41.97	44.14	100
Woman_darkhair	48.35	43.69	47.77	46.87	100
Clock	48.91	48.67	46.85	47.59	100
Chemical plant	42.64	43.83	42.74	49.65	100
Walter cronkite	47.72	47.25	48.65	46.39	100
Average	45.13	45.10	46.20	45.46	100

### Comparison

The comparative analysis of the proposed scheme against existing watermarking techniques is detailed in Table 7. The techniques compared include widely adopted methods such as DWT, IWT, and LSB embedding and block mapping, among others, highlighting the diversity of approaches in the field. Each technique was assessed based on its ability to achieve tamper localization, image recovery, and the Peak Signal-to-Noise Ratio (PSNR) of both watermarked and recovered images.

A key observation is that the proposed scheme achieves the highest PSNR for watermarked images (45 dB) and recovered images (43 dB), surpassing other approaches, such as those in Ref.^35^ and Ref.^26^. This significant improvement indicates that the proposed method maintains high visual quality for watermarked images and effectively restores the original image after tamper detection, ensuring minimal distortion. In contrast, some existing methods, such as Ref.^36^ and Ref.^37^, either lack recovery capability or report lower PSNR values, compromising their effectiveness in applications requiring high fidelity.

The robustness of the proposed scheme can be attributed to its use of Discrete Wavelet Transform (DWT), which efficiently balances the trade-off between imperceptibility and robustness. Unlike block mapping methods, which suffer from lower PSNR values due to block-wise processing, DWT preserves image details while embedding the watermark, making it more suitable for tamper localization and recovery.

Furthermore, including the 2D Lift Wavelet method in Ref.^38^ demonstrates a noteworthy improvement in recovered image quality (PSNR of 40 dB). However, the proposed scheme outperforms even this advanced method, emphasizing its superior design and implementation. Similarly, while techniques like LSB embedding (Ref.^35^) provide simplicity, their lower PSNR (33.46 dB) and lack of recovery capabilities make them less reliable for applications demanding higher security and accuracy.

The table also highlights methods such as Ref.^25^, which differentiate between regions of interest (ROI) and non-interest (RONI), achieving a competitive PSNR for both watermarked (45 dB) and recovered images (42 dB). However, the additional complexity of ROI/RONI segmentation introduces overheads that are avoided in the proposed approach, making it more practical for real-time applications. The work ^13^ uses IWT-SVD and achieved 50.67 dB, where only embedding and extraction are done without any tamper detection and recovery.

Overall, the proposed scheme demonstrates its capability to localize tampering and recover images while maintaining superior PSNR values, setting a new benchmark in image tampering detection and restoration.

**Table 7:** Comparison of proposed scheme with related watermarking schemes.

Schemes	Technique	Tamper localization	Recovery	PSNR WI	PSNR RI
^35^	LSB Embedding	Yes	Yes	33.46	–
^25^	ROI and RONI	Yes	Yes	45	42
^39^	DWT	Yes	Yes	42	32
^36^	DWT	No	No	40	–
^37^	DWT	Yes	No	41	–
^38^	2D Lift Wavelet	Yes	Yes	43	40
^24^	IWT	Yes	Yes	40	-
^26^	Block Mapping	Yes	Yes	39	35
^13^	IWT	No	No	50.67	–
Proposed	DWT	Yes	Yes	45	43

## Conclusion

This paper presents a robust method for image tampering detection and recovery. The proposed approach authenticates each image block of size 4$\times$4 by utilizing DWT coefficients. The K-means clustering algorithm addresses each 2$\times$2 sub-block of the image to enhance the recovery process. The method integrates fragile watermarking to embed authentication and recovery data into the spatial domain of the original image. This integration is crucial for effective tamper detection, with block dependencies providing more accurate tampering identification. Using the K-means clustering algorithm significantly enhances the recovery performance compared to existing methods, demonstrating superior tampering detection and image restoration results. However, although Schur decomposition and ABBs ensure accurate tamper detection and recovery, the technique may require further optimization to enhance performance under more complex and diverse tampering scenarios.

## Data Availability

The dataset analyzed during the current study is available in the ImageProcessingPlace repository: https://www.imageprocessingplace.com/ and SIPI Image Database: https://sipi.usc.edu/database/.
